# The effect of an information intervention on the career commitment of medical students: evidence from a randomized experiment

**DOI:** 10.3389/fmed.2023.1101993

**Published:** 2023-05-22

**Authors:** Chunqing Li, Xiaoyang Ye, Chen Yu, Hongbin Wu

**Affiliations:** ^1^Institute of Medical Education/National Center for Health Professions Education Development, Peking University, Beijing, China; ^2^Annenberg Institute for School Reform, Brown University, Providence, RI, United States

**Keywords:** career commitment, information intervention, role modeling, behavioral economics, difference-in-differences

## Abstract

**Introduction:**

The needs-based shortage of healthcare workers is severe worldwide and it would be exacerbated if many medical students switch to other careers after graduation. Maintaining and improving the career commitment of medical students, which could be a feasible, effective, and scalable way to reduce the attrition rate, is essential in medical education. We designed a randomized experiment to test whether an information intervention based on role modeling could enhance medical students’ career commitment.

**Methods:**

In the randomized experiment, the sample (*N* = 36,482) was divided into the treatment group (*N* = 18,070) and the control group (*N* = 18,412). The intervention information consisted of image-text messages on Zhong Nanshan, who is an inspiring role model for he went to the frontline of COVID-19 in the most critical circumstances and received praise and affirmation from the public. Α difference-in-differences model was employed to identify the effect of the information intervention. Heterogeneous treatment effects were identified using sub-sample analyses.

**Results:**

The results showed that the information intervention statistically significantly reduced medical students’ dropout intention by 2.7 percentage points (95% CI: −0.037 to −0.016, *t* = −4.95, *p* < 0.001), equivalent to 14.6% of the control group mean. This estimate indicates that the information intervention could significantly increase the career commitment of medical students. Finally, male and senior students were influenced more than their female and junior counterparts, which can be explained by their relatively high dropout intention.

**Conclusion:**

Role model-based information intervention improves the career commitment of medical students. The underlying behavioral model is that, when students use a role model as their reference point, they consider dropout as a substantial welfare loss. Role modeling is an effective way to improve the career commitment of medical students, especially for males and senior students.

## Introduction

To fulfill the United Nations’ Sustainable Development Goals and achieve universal health coverage, human resources in healthcare are the priority (1). However, there is a great demand for healthcare workers globally. A shortage of 6.4 million physicians was recorded worldwide in 2019 (2) and predictions indicate that it would even hit 14 million by 2030. Current trends in health worker supply could not cover this shortage (1). The number of medical students cultivated in higher education institutions now is barely enough for the demand of healthcare labor market, let alone some decide to switch to other careers after graduation, which worsens the situation.

Maintaining and improving the career commitment of medical students may be a feasible, effective, and scalable way to reduce the shortage (3–5). Career commitment is the commitment of an individual to a career or profession, reflecting their desire and preference for their profession, which is an attitude and determination to continue engaging in the chosen career (5, 6). It has also been operationalized regarding the reluctance to leave the professional role (5, 7). Therefore, improving career commitment is the priority of medical education since it can help accelerate progress toward universal health coverage. Previous studies on career commitment, mainly used qualitative methods, have examined questions like career commitment formation (8–10), career commitment change in clinical environments (5, 11), the role of career commitment in career choice (11), and burnout (12). The factors that affect career commitment have also been analyzed (5, 9, 13–15), one of which with high importance is the role model (10, 15, 16).

According to social learning theory, learning from role models is an effect way to improve individuals’ ability to deal with a specific task or activity (17). Role models personify the ideal selves people aspire to be by manifesting clinical attributes, personal qualities and positive professional characteristics, and they can be emulated by others, especially young people (18–21). It has been proved that role modeling is a powerful, underexploited, and deliberate teaching intervention and strategy (22–24). This type of intervention originates from the prospect theory in behavioral economics. Prospect theory suggests that people have reference-dependent preferences, and the utility gained from decision-making under uncertainty depends on the perceived gain or loss relative to the reference point. The theory also proposes the principle of loss aversion, which states that the positive utility from the same amount of gain is less than the negative utility from the same loss (25, 26). A role model serves as a reference point. Individuals compare themselves with the role model, and utility loss occurs when their behavior deviates from the corresponding one of the role models. Research related to role models and commitment has focused on the function of role modeling in the formation of professional attitudes (27), professionalism (28, 29), professional identity (10, 16), and career choices (30). The effect of role modeling on career commitment was found to be heterogeneous among individuals (15). Survey data were used in some previous research (31), yet fewer studies have adopted experimental methods to identify the effects of role model-based interventions on career commitment, which provides an opportunity for this study. According to social learning theory and prospect theory, we hypothesize that role-model based information interventions can improve the career commitment of medical students.

Similar to other countries, China also has a high attrition rate among medical major graduates and physicians (32). This study aims to investigate practicable information intervention to improve the career commitment of Chinese medical students in a low-cost and predictable manner. Our results are expected to not only alleviate the shortage of human resources in China but also provide important evidence-based implications for other countries. The research questions this study addressed are as follows: (1) whether an information intervention based on a role model boosts the career commitment of medical students, and (2) whether the information intervention has heterogeneous effects on different medical students. The exploration of these questions will advance our understanding of the effect of role-model based information intervention on the career commitment of medical students.

## Methods

A randomized experiment, designed in line with the CONSORT guidelines, was implemented in China between June 10 and July 10, 2021. We collected the data through the “National Center for Medical Education Development Information and Data Platform” in the form of an online survey (33). Ethical approval (IRB00001052-20069) was received from the Institutional Review Board of Peking University, Beijing, China. The electronic informed consent was obtained at the beginning of the survey from all participants included in the study. Students voluntarily participated in and were informed that their data would be kept anonymous and confidential.

### Inclusion and exclusion criteria

In this experiment, we recruited participants from 93 randomly selected medical higher education institutions (out of 165 in total). Inclusion criteria included respondents who were full-time undergraduate students in those 93 institutions and majored in clinical medicine. Exclusion criteria included that the participants did not agree to provide the online consent. We also excluded foreign students, students from Hong Kong, Macao, and Taiwan (Republic of China), considering that their culture, training process, and future career choice are very different from that of clinical medical students in Chinese mainland.

### Sample

**Figure 1:**
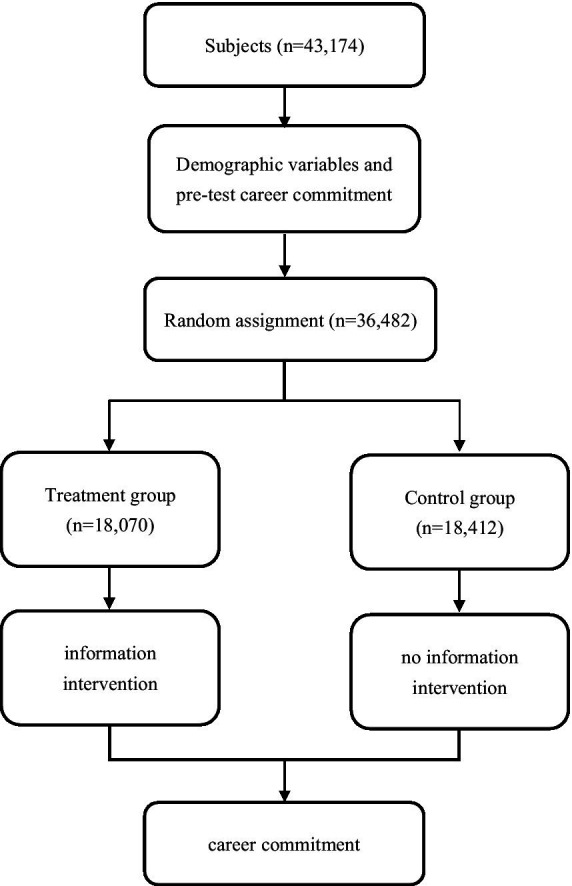
Flow chart of the experiment.

The minimum sample sizes needed for the experiment were calculated according to Bloom and Dong, and Maynard (34, 35). Commonly, set *α*, the probability of making Type I error, equal to 0.05 and power equal to 0.8 (35). The minimum sample size was estimated to be 35,663. The number of institutions offering the undergraduate Medical Education program in China is 165, with 44.8% located in the eastern provinces, 29.7% in the center, and 25.5% in the west (36). Based on the minimum sample size and the distribution of medical institutions in China, a total of 43,174 undergraduate medical students were sampled from 93 institutions in 30 provinces, including 41 institutions (44.1%) located in the east, 27 institutions (29.0%) in the center and 25 institutions (26.9%) in the west. The geographic distribution of the selected sample was correspondingly balanced with that of China; hence the results of the study is reasonably representative. A total of 36,482 medical students participated in the experiment, with a response rate of 84.5% (see Figure 1).

### Intervention

#### Pre-intervention survey

In the pre-intervention stage, characteristics of the subjects were collected through an online survey, including pre-intervention career commitment, demographic information (gender, ethnicity, Communist Party of China members, and only child status), family background (having a doctor parent, father’s education, mother’s education, high-income families, father’s International Socio-Economic Index, mother’s International Socio-Economic Index), educational background (National College Entrance Examination scores, expectation, career planning). The questionnaire used is self-built. We designed the survey with references to the opinions of experts (in medical education, social science surveys, clinical medicine, and other closely related fields) and stakeholders (students). Before the formal implementation, we selected some medical students for the pre-test, so as to make the information collected by the survey accurately expressed and validated in content.

#### Intervention

The sample was randomly assigned into two groups by the online survey platform randomization algorithm. The treatment group received the information intervention, while the control group did not. The study personnel member who independently created the randomization process did not involve in participants recruitment or data analysis. The participants did not know the intervention content before participation and they only reached the content of their group and were unaware of what happened in the other group after randomization. The delivery method was that we inserted intervention information into the survey, containing the image and text on the role model of Zhong Nanshan, a public face of China’s efforts in the fight against COVID-19. Zhong Nanshan went to the front line of the battle against COVID-19 and spread praise and affirmation of the pro-social behaviors of healthcare workers by mainstream social media. Our intervention information has been validated by Ye et al. (2022) who used the text-image to identify the effect of nudge information on students’ post-pandemic dropout intention (15).

In the post-intervention stage, career commitment was measured again. In our experimental design, the intervention and the measurement of career commitment were displayed sequentially on different screens. There was a time lag between the intervention and the measurement outcome (see Figure 1).

### Measurement and collected data

Career commitment was the dependent variable measured by a five-point Likert Scale question “I want to become a doctor after graduation.” Those who selected options “strongly disagree” and “disagree” were coded as having a dropout intention (i.e., career dis-commitment). Medical students with a dropout intention had lower career commitment.

Treatment was used as the independent variable. We assign treatment values of 1 for individuals in the treatment group and of 0 for individuals in the control group.

The control variables included gender, ethnicity, CPC members (Communist Party of China), only child, NCEE score (National College Entrance Examination), doctor parent, father’s education, mother’s education, high-income family, father’s ISEI (International Socio-Economic Index), mother’s ISEI, expectation, and career planning. CPC members variable equaled 1 for individuals who were CPC members. The NCEE score variable was standardized. Most provinces used the national unified college entrance exam design with a total score of 750. For provinces where the total score was not 750, the original NCEE scores were converted to 750. The doctor-parent is a dummy variable that took value 1 if either parent was a healthcare worker and 0 otherwise. The high-income family variable was also a dummy variable which took value 1 when the family had an income higher than 150,000 RMB in the last year. Father’s (mother’s) ISEI measured whether the father’s (mother’s) ISEI was higher than the median of the father’s (mother’s) ISEI. The expectation variable corresponded to the question, “Was the college admission result lower than your expectation?.” In addition, the career planning variable corresponded to the survey item “Have you participated in a career planning program in high school?,” which equaled 1 if the “yes” option was chosen.

### Data analysis

We first performed a balance test to validate the random assignment. Each control variable was regressed on the treatment variable to report the t-test results. Then, the treatment variable was regressed on all controls to report the joint F test results.

Next, a difference-in-differences (DID) model was employed to identify the effect of the information intervention, as follows.


(1) $$Y_{i,t}=\beta Treat_{i}\times Post_{t}+{\left(X_{i}\times Post_{t}\right)}^{’}\gamma +\eta_{i}+\tau_{t}+\varepsilon_{i,t}$$

Eq. (1) identifies the causal effect of an information intervention on medical students’ dropout intention using the DID model. Let *i* index the individual and *t* index the period; $Y_{i,t}$ denotes the intention of individual *i* to drop out of medicine in period *t*, which measures career commitment; $Treat_{i}$ denotes whether individual *i* was assigned to the treatment group; $\;Post_{t}$ denotes the period dummy variable, which equals 1 at *t*, and $\varepsilon_{i,t}$ is the error term; β captures the effect of the information intervention on students’ career commitment, which is the main interest of this study; $X_{i}$ is a series of predetermined controls, including gender, ethnicity, CPC members, only child, NCEE score, doctor parent, father’s education, mother’s education, high-income families, father’s ISEI, mother’s ISEI, expectation and career planning; $\eta_{i}$ and $\tau_{t}$ are individual fixed effects and time fixed effects, respectively. According to Bertrand et al. (37), standard errors should be clustered to the individual level due to the correlation of the same individual’s error terms at different times. Therefore, the standard errors were clustered to the individual level.

Finally, the heterogeneity effects of the information intervention were examined. In each subsample, Eq. (1) was used to identify the heterogeneity effects.

## Results

### Characteristics of the sample

A total of *N* = 36,482 medical students were included in the study sample. About 42% of students were male and over 13% of students were CPC members. The sample contained over 37% only-child. The average NCEE score of the sample was 547.8. A subset of *N* = 18,412 was assigned in the control group and N = 18,070 in the treatment group. About 10% of the student’s parents were healthcare workers. The average years of schooling of the students’ fathers and mothers were 11 years and 10 years, respectively. More than 30% of students’ parents had a high ISEI. Nineteen percent of students were from high-income families. Thirty-five percent of students believed the college admission results were lower than their expectations. Less than 30% of students took part in career planning programs in high school (see Table 1).

**Table 1:** Descriptive statistics and balance test.

	(1)	(2)	(3)	(4)	Full sample	Control group	Treatment group	T –C
Preintervention dropout intention	0.167	0.165	0.169	0.004
	[0.373]	[0.371]	[0.375]	(0.006)
Male	0.420	0.419	0.422	0.004
	[0.494]	[0.493]	[0.494]	(0.007)
Han ethnicity	0.871	0.871	0.870	−0.001
	[0.335]	[0.335]	[0.336]	(0.005)
CPC members	0.127	0.129	0.125	−0.003
	[0.333]	[0.335]	[0.331]	(0.005)
Only child	0.378	0.376	0.379	0.002
	[0.485]	[0.484]	[0.485]	(0.007)
NCEE score	547.772	547.282	548.273	0.991
	[60.818]	[60.746]	[60.889]	(0.901)
Doctor parent	0.115	0.115	0.116	0.001
	[0.320]	[0.319]	[0.320]	(0.005)
Father’s education	10.706	10.719	10.693	−0.027
	[3.837]	[3.844]	[3.829]	(0.057)
Mother’s education	9.616	9.610	9.621	0.010
	[4.279]	[4.257]	[4.302]	(0.063)
High-income family	0.135	0.131	0.138	0.007
	[0.341]	[0.338]	[0.345]	(0.005)
Father ISEI	0.343	0.343	0.343	−0.000
	[0.475]	[0.475]	[0.475]	(0.007)
Mother ISEI	0.322	0.317	0.326	0.009
	[0.467]	[0.465]	[0.469]	(0.007)
Expectation	0.346	0.341	0.350	0.009
	[0.476]	[0.474]	[0.477]	(0.007)
Career planning	0.299	0.300	0.298	−0.002
	[0.458]	[0.458]	[0.457]	(0.007)
*N*	36,482	18,412	18,070	

(1) CPC members means the members of Communist Party of China. (2) NCEE score means National College Entrance Examination score. (3) ISEI means International Socio-Economic Index.

### Balance test

Before identifying the effect of the information intervention, a balance test was performed. No statistically significant differences were detected between the treatment group and the control group regarding dropout intention pre-intervention (*p* = 0.518), gender (*p* = 0.622), Han ethnicity (*p* = 0.841), CPC members (*p* = 0.500), only child (*p* = 0.742), NCEE score (*p* = 0.271), doctor parent (*p* = 0.821), fathers’ education (*p* = 0.640), mothers’ education (p = 0.821), high-income family (*p* = 0.186), fathers’ ISEI (*p* = 0.958), mothers’ ISEI (*p* = 0.178), expectation (*p* = 0.212) and career planning (*p* = 0.772). Regression of all variables in Table 1 using the treatment dummy variable resulted in an *F* value of 0.69 and a *p* value of 0.79, indicating that the groupings were unrelated to observable individual characteristics.

### The average effect of the information intervention

Table 2 suggested that the information intervention statistically significantly reduced medical students’ dropout intention rate by 2.7 percentage points (SE = 0.005, 95% CI: −0.037 to −0.016, *t* = −4.95, *p* < 0.001), equivalent to 14.6% of the control group mean. The estimates remained consistent after controlling for the interaction of a range of observable control variables and a post dummy variable, suggesting the information intervention significantly boosted career commitment in medical students. While the measurement of outcome variable was on the next screen of the information intervention in our experiment and the treated students spent some time reading the intervention information before reporting their career commitment, this treatment effect represents an immediate behavioral response to the role model-based information.

**Table 2:** Effects of information intervention on medical students’ career commitment.

	(1)	(2)
	dropout intention	dropout intention
Treat*post	−0.027^***^	−0.027^***^
	(0.005)	(0.005)
Controls*post	NO	YES
Individual FE	YES	YES
Time FE	YES	YES
N	36,482	36,482
R2	0.779	0.779

(1) Control variables include gender, ethnicity, CPC members, only child, NCEE score, doctor parent, father’s education, mother’s education, high-income family, father’s ISEI, mother’s ISEI, expectation, and career planning. (2) Robust standard errors are clustered at the individual level. (3) *** *p* < 0.01, ** *p* < 0.05, * *p* < 0.1.

### The heterogeneity effect of the information intervention

**Figure 2:**
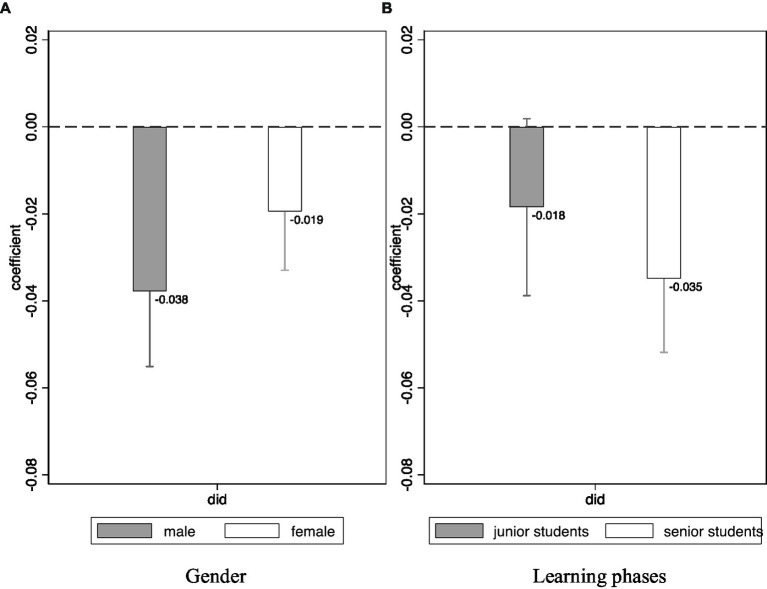
The heterogeneity effect of information intervention.

The influence of the information intervention on different students was then analyzed, and the results are shown in Figure 2. The results of the heterogeneity analysis indicated that the treatment effect of the information intervention on male students was *β* = −0.038 (SE = 0.001, 95% CI: −0.055 to −0.020, *t* = −4.28, *p* < 0.001) and *β* = −0.019 (SE = 0.007, 95% CI: −0.033 to −0.006, *t* = −2.83, *p* = 0.005) on female students. Thus, the information intervention influenced the career commitment of male students more than that of female students. Additionally, the influence of information intervention on junior students was *β* = −0.018 (SE = 0.010, 95% CI: −0.039 to 0.002, *t* = −1.78, *p* = 0.075) and *β* = −0.035 (SE = 0.009, 95% CI: −0.052 to −0.018, *t* = −4.04, p < 0.001) on senior students, meaning that senior students benefited more from the intervention.

## Discussion

This study designed a randomized experiment and used a DID model to estimate the effect of an information intervention on dropout intention in medical students. The information intervention, a combination of text and image, was based on a role model in the fight against the COVID-19 pandemic. The results indicated that the information intervention significantly increased the career commitment of all medical students, with the male and senior students being influenced more than their female and junior counterparts.

The results demonstrated that role modeling is an effective teaching strategy to boost career commitment, which agrees to Wang and his colleagues’ research (12). Role models provide inspiration and a comparing standard for students. Role models model how to achieve tasks and transmit valuable information. The behavior of role models amplifies students’ belief in reaching their goals. Students compare themselves with role models and follow their behaviors. Therefore, the role models serve as the reference point, and students revised their views by observing the role model and reflecting on their behavior (38). In this case, dropout was considered as a loss for them, which reduced their utility due to the loss aversion principle; therefore, students molded themselves to the role model’s behavior. Role models encouraged students to actively participate in the medical profession. The results of the present study are consistent with previous research’s, indicating that medical students are inspired when observing pro-social behaviors such as respect, compassion and empathy (16).

The information intervention was revealed to have had a greater effect on male students than female students. Studies have shown that male students are more likely to drop out of school than female students (39–43). This study showed that male students had a higher dropout intention than female students before the intervention, suggesting the presence of gender differences in initial career commitment. The informational intervention served to narrow the gender differences in career commitment.

Moreover, the results demonstrated that senior medical students would benefit more from the intervention than junior medical students. A previous study found that the career commitment of senior medical students gradually weakens (5, 14). In our sample, the dropout intention in senior medical students was higher than junior medical students. This may be explained by the fact that senior medical students are exposed to greater interpersonal pressure, complex doctor-patient relationships, and heavy academic and practice loads due to clinical medical education and clerkship rotation, which may reduce their determination and enthusiasm to continue practicing medicine. The educational student engagement in senior medical students is also lower than that in junior medical students (36). Educational student engagement is positively correlated with learning outcomes, and students who struggle academically are more likely to drop out (44). Therefore, information intervention is crucial in helping students with higher dropout intentions increase their career commitment and significantly improve their well-being, and thus make better career choices.

This study also indicates that the career commitment of medical students changed over time. Students experience a dynamic, gradual process during medical education, such as the reduction in career commitment of students in advanced degree stages receiving clinical medical education and clerkship rotation. Educational planners should adopt effective education strategies to maintain and enhance career commitment during medical education. Role modeling positively affects career commitment, especially for medical students with low career commitment. Therefore, role modeling can be integrated into the course and daily life of medical students to build up their career commitment and help them make better career choices, which will further increase human resources for health supply and promote social well-being.

Our results provide evidence-based support for the design and implementation of similar low-cost information interventions to improve medical students’ communication with chronic patients. For example, patients, patients’ family, and the medical and psychological team are the stakeholders of a comprehensive care system in the management of the hemophilic children. Large educational efforts and on-going medical and psychological support need to be offered to families (45). The information intervention based on role models who had rich experience in communication with patients may be a possible teaching method in the intensive training and education.

### Limitations

The role model presented is based on informational interventions and is a light-touch, low-cost approach. Combining role models with different degrees of exposure may have more sustainable and long-term effects on medical students’ career commitment. The actual commitment behaviors to the career of individuals might be used to measure career commitment more than intention. Our results speak to the immediate impacts when students are presented with new information. Tracking the participants over a longer time period and finding out whether or not the intervention has any effect on actual attrition would be an important direction for future work.

## Conclusion

A randomized experiment was implemented to test the effect of an information intervention on the medical students’ career commitment. The information intervention approach presented in this study is based on a role model, showing medical students the pro-social behavior of healthcare workers in the fight against the COVID-19 pandemic. The positive effect of the information intervention on medical students’ career commitment was identified, and an analysis of this effect on its potential heterogeneous impact on medical students based on their gender and learning stage was also performed. The findings reported in this study enrich the evidence that information intervention could improve the career commitment of medical students in developing countries and bring about possible solutions to alleviate the global shortage of human resources in healthcare.

## Notes on contributors

Chunqing Li, PhD, is a postdoc at National Centre for Health Professions Education Development/Institute of Medical Education, Peking University. She holds a PhD from Beijing Normal University focusing on health professions education. She has published articles in Comparative Economic & Social Systems, Social Sciences of Beijing and other top-tier journals. She has also translated book such as Essential Skills for a Medical Teacher.

Xiaoyang Ye, PhD, was a postdoctoral researcher in education policy in the Annenberg Institute for School Reform at Brown University. He holds a PhD from University of Michigan focusing on the economics of education. He has published articles in BMC Medical Education, Economics of Education Review, Journal of Economic Behavior & Organization and other top-tier journals.

Chen Yu, PhD, is an lecturer at National Centre for Health Professions Education Development/Institute of Medical Education, Peking University. She holds a PhD from Peking University focusing on medical education. She has published articles in Public Health, Medical Education and other journals. She launched the China Medical Student Survey.

Hongbin Wu, PhD, is an assistant professor at National Centre for Health Professions Education Development/Institute of Medical Education, Peking University. He holds a PhD from Peking University focusing on health professions education. He has published articles in Medical Education, Computers & Education, Medical Teacher and other top-tier journals. He has also translated books such as *A Practical Guide for Medical Teachers* and *Researching Medical Education*. He has worked as an investigator in many national-level research projects and launched the China Medical Student Survey.

## Data availability statement

The raw data supporting the conclusions of this article will be made available from the corresponding author upon reasonable request.

## Ethics statement

The studies involving human participants were reviewed and approved by PU IRB (approval number: IRB00001052-20069). The patients/participants provided their written informed consent to participate in this study.

## Author contributions

CL, XY, CY, and HW designed the study and wrote the manuscript. HW performed the experiments. CL analyzed the data. All authors contributed to the article and approved the submitted version.

## Funding

The research reported here was supported by a grant from the National Natural Science Foundation of China (72174013). None of these funding bodies has exerted any influence on the study design and collection, analysis, interpretation of data, or on writing the manuscript.

## Conflict of interest

The authors declare that the research was conducted in the absence of any commercial or financial relationships that could be construed as a potential conflict of interest.

## Publisher’s note

All claims expressed in this article are solely those of the authors and do not necessarily represent those of their affiliated organizations, or those of the publisher, the editors and the reviewers. Any product that may be evaluated in this article, or claim that may be made by its manufacturer, is not guaranteed or endorsed by the publisher.
